# Antibodies induced by immunization with a hypoallergenic mutant of the major fish allergen Cyp c 1 inhibit allergic symptoms in a mouse model of fish allergy

**DOI:** 10.1186/2045-7022-3-S3-O22

**Published:** 2013-07-25

**Authors:** AC Gstoettner, U Baranyi, M Focke-Tejkl, I Swoboda, F Stolz, T Wekerle, B Linhart, R Valenta

**Affiliations:** 1 Pathophysiology and Allergy Research, Medical University of Vienna, Vienna, Austria; 2 Department of Surgery, Medical University of Vienna, Vienna, Austria; 3 Biomay, Vienna, Austria

## Background

Fish allergy is one of the most frequently occurring allergies to animal-derived food and may cause severe anaphylactic reactions. Currently allergen-specific immunotherapy for fish allergy is not available as it may induce severe side-effects. A hypoallergenic mutant of the major carp allergen Cyp c 1 (mCyp c 1) has recently been developed for the treatment of IgE-mediated fish allergy. We analysed the effect of antibodies induced by immunization with mCyp c 1 on allergic symptoms in a mouse model of fish allergy.

## Methods

C3H/HeJ mice were sensitized to Cyp c 1 by intragastric gavage or subcutaneous immunization using Cholera toxin or aluminium hydroxide as adjuvans, respectively. Rabbits were immunized with the recombinant mCyp c 1. Antibody responses to mCyp c 1 and wild-type Cyp c 1 were investigated by ELISA using 7 overlapping Cyp c 1-derived synthetic peptides. In order to study if mCyp c 1-specific antibodies can protect, Cyp c 1-sensitized mice received mCyp c 1-specific or control antibodies before they were challenged orally with Cyp c 1.

## Results

Intragastric sensitization of C3H/HeJ mice induced Cyp c 1-specific IgE antibodies, which did not bind to Cyp c 1-derived peptides, indicating sensitization to conformational epitopes. Rabbit anti-mCyp c 1-specific IgG antibodies recognized the wildtype Cyp c 1 allergen and also reacted with peptide epitopes. They were able to block human and mouse IgE-binding to rCyp c 1 *in vitro*. *In vivo* administration of mCyp c 1-specific IgG antibodies reduced allergic symptoms after oral allergen challenge demonstrating that allergen-specific IgG antibodies can protect against food allergy.

## Conclusion

Vaccination with mCyp c 1 induces IgG antibody responses, which can protect against fish allergy in a murine model.

This work was supported by the FAST project of the FP7 of the European Union.

## Disclosure of interest

None declared.

